# How is Ambulatory Electrocardiogram Predictive of Stroke in Atrial Fibrillation Patients?

**DOI:** 10.1155/2022/7619669

**Published:** 2022-10-10

**Authors:** Xiuping Zhuo, Meinv Huang

**Affiliations:** Affiliated Hospital of Putian University, No. 999 Dongzhen East Road, Licheng District, Putian, Fujian, China

## Abstract

**Background:**

Atrial fibrillation (AF) is a significant stroke risk factor. Further research is needed to clarify whether higher atrial fibrillation burden (AFB) link to the elevated risk of ischemic embolism, and how AF burden could combine with CHA_2_DS_2_-VASc score to improve the anticoagulation strategy. We aim to evaluate if the AF burden characterized using 24-hours Holter ECG monitoring is associated with the risk of ischemic stroke.

**Methods:**

This cohort study enrolled 210 Holter ECG monitoring detected atrial fibrillation patients. The burden of atrial fibrillation was defined as the percentage of time in atrial fibrillation during the monitoring period, and the AF burden and CHA_2_DS_2_-VASc score were compared between patients with and without thromboembolic outcomes. Multivariate regressions were conducted to estimate the predictors of thromboembolic outcomes.

**Results:**

Eighteen thromboembolic events occurred within a median follow-up of 11.39 months. Patients with ischemic stroke had higher CHA_2_DS_2_-VASc scores but not higher AF burden. After adjusting for age, hypertension, diabetes, anticoagulation, antithrombotic therapy, AF burden, and AF with higher CHA_2_DS_2_-VASc score was associated with increased risk for ischemic stroke (hazard ratio (HR), 15.17). CHA_2_DS_2_-VASc score > 4.5 was a predictor of significantly higher risk of future stroke (AUC 0.92).

**Conclusions:**

In Holter ECG monitoring detected AF, AF burden does not significantly impact the subsequent risk of stroke; whereas, CHA_2_DS_2_-VASc scoring is still a robust predictor of stroke risk. This may illustrate that once AF is detected from Holter ECG monitoring, underlying risk factors appear to be more predictive of subsequent stroke risk than atrial fibrillation burden.

## 1. Introduction

Atrial fibrillation (AF) is a serious public health problem because of its increasing prevalence in the aging population and it is considered a potent risk factor for stroke. Current guidelines regard AF as a binary, present or absent, with the decision to anticoagulant therapy only by CHA_2_DS_2_-VASc based on clinical characteristics, regardless of AF pattern or burden. However, the development of monitoring techniques opens the door to a long-term pattern of atrial fibrillation monitoring and better defines the importance of the burden of atrial fibrillation over time. The atrial fibrillation burden (AFB) is usually defined as the percentage of time that atrial fibrillation occurs over a sufficiently long monitoring period [1]. Some trials show consistently lower stroke risk in paroxysmal AF versus persistent AF but similar risks in other studies. Further research is needed to clarify whether a higher atrial fibrillation burden is linked to an elevated risk of ischemic embolism, and the threshold of AF burden that warrants anticoagulation. In our study, we aimed to evaluate the association between AFB and ischemic stroke using noninvasive 24-hours Holter ECG monitoring, and to explore the potential of AFB as an indicator of stroke event and further supplement of CHA_2_DS_2_-VASc scoring system.

## 2. Methods

We performed a cohort study consisting of 220 patients with atrial fibrillation that underwent 12-lead 24-hours Holter ECG monitoring from January 2020 to December 2020 at Affiliated Hospital of Putian University. The diagnosis of atrial fibrillation and related quantitative data were reconfirmed by an electrophysiologist after an automated system analysis. For patients who met the atrial fibrillation inclusion criteria, AF burden was defined as the cumulative percentage of time met AF detection criteria in the given Holter ECG monitoring day and AF frequency was calculated. Acute ischemic stroke was defined as cerebral infarction in the clinical record, following the diagnostic criteria of China guidelines for the diagnosis and treatment of acute ischemic stroke 2014. Patients with valvular AF, thyroid diseases were excluded. We also excluded patients who had completed radiofrequency ablation for atrial fibrillation. The follow-up was conducted by telephone survey and electronic medical record inquiry with an average follow-up time of (11.39 ± 6.00) months until June 30, 2021. Basic demographic characteristics, comorbidities, drug use, and major clinical events including all causes of hospitalization, death, new embolism, and massive bleeding were recorded, with a new onset of ischemic stroke as the primary outcome. 10 patients were unreachable and lost follow-up, with a follow-up rate of 95.45%, and the remaining cases were divided into the control group (192 cases) and stroke group (18 cases) according to whether acute ischemic stroke occurred during the follow-up.

The baseline characteristics and the outcomes were compared and grouped according to the presence of new stroke using the *t*-test, Kruskal–Wallis, one-way ANOVA chi-square, and Fisher's exact tests were appropriate. We calculated the CHA_2_DS_2_-VASc score, then evaluated hypertension, diabetes, antiplatelet therapy, anticoagulation therapy, and CHA_2_DS_2_-VASc score as categorical variables, meanwhile AF burden and AF frequency as continuous variables. These variables were evaluated for the correlation with stroke using binary logistic regression analysis model. Finally, the predictive value of the CHA_2_DS_2_-VASc score was further evaluated using the c-statistic of receiver operating characteristic (ROC) analysis. All the above analyses were performed using IBM SPSS vs. 26, and statistical significance was set at a two-tailed *P* < 0.05.

## 3. Results

Among the 210 atrial fibrillation patients enrolled in our study, 18 thromboembolic events occurred within a median follow-up of 11.39 months after the Holter ECG monitoring. 10 cases were hospitalized for “cerebral infarction” within 1 month of Holter ECG monitoring, and 4 patients with a previous ischemic stroke had a recurrent stroke at 10th, 12th, 4th, and 6th months. Baseline characteristics of AF patients with and without stroke are shown in Table 1. Patients in the stroke group were statistically older than the control group, with an average age of 75 years in the stroke group versus 70 years old in the control group. The proportion of hypertension history and diabetes history in the stroke group was 77.8% and 33.3%, respectively, higher than those in the control group (38.5% and 12.5%), with statistical significance (*P* < 0.05). Among the 192 patients in the control group, 31 patients took rivaroxaban regularly and 87 patients took warfarin, of which 8 patients did not routinely monitored for INR and INR fluctuated drastically in other 10 patients. All 18 stroke patients had CHA_2_DS_2_-VASc scores ≥ 2 and received anticoagulant therapy following the prescription, of which 5 patients took rivaroxaban and 13patients took warfarin, but 3 patients did not routinely monitored for INR and 2 patients had wildly fluctuating INR. Anticoagulation therapy was considered effective when patients took rivaroxaban or were regularly prescribed warfarin with proper international normalized ratio (INR). The utilization rate of anticoagulant drugs in stroke patients was higher than that of the control group (61.5%), while the utilization rate of antiplatelet aggregation drugs in the control group was higher than that in the stroke group (26.0% vs. 0.00%, respectively), with statistical significance (*P* < 0.05). But there was no significant difference in the proportion of anticoagulant efficacy between the two groups, listed in Table 1. There were no statistically significant differences in gender, coronary heart disease history, peripheral artery disease history, heart failure history, statins, amiodarone and *ß*-blocker usage between the two groups.

**Figure 1:**
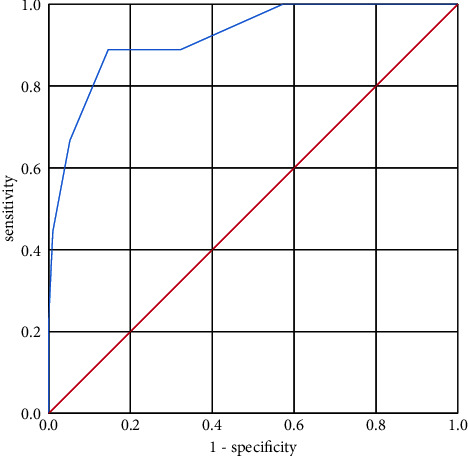
ROC curve of CHA_2_DS_2_-VASc score for stroke event.

Notably, patients with ischemic stroke had higher CHA_2_DS_2_-VASc scores but not higher AF burden. Meanwhile, there was no significant difference in the AF pattern and AF frequency between the two groups, as shown in Table 2. Subgroup analysis showed that the proportion of patients with CHA_2_DS_2_-VASc scores 0, 1, and ≥2 in the control group was 9.4%, 13.5%, and 77.1%, respectively, while in the stroke group all patients had CHA_2_DS_2_-VASc scores ≥ 2. Binary logistic regression analysis showed that after adjusting for age, hypertension, diabetes, anticoagulation, antithrombotic therapy, AF burden, and AF with higher CHA_2_DS_2_-VASc score was associated with increased risk for ischemic stroke (hazard ratio (HR), 15.17); 95% confidence interval (CI), 3.77–61.00; *P* < 0.01). The results are shown in Table 3 below. As shown in Figure 1, CHA_2_DS_2_-VASc score > 4.5 was a predictor of significantly higher risk of future stroke (AUC 0.92, sensitivity 0.89, specificity 0.85, *P* value < 0.01).

## 4. Discussion

Atrial fibrillation (AF), with an annual incidence of 5.38 per 1,000 person-years [2, 3], is one of the most common clinical arrhythmias. A large body of literature has documented that atrial fibrillation is a potent risk factor for stroke, and early detection may allow timely therapeutic intervention. As a supplement to qualitative diagnosis, the application of long-term continuous ECG monitoring technology brought the concept of a quantitative diagnosis of atrial fibrillation and also led to the study on the relationship between atrial fibrillation burden. But there remains no clear consensus on whether the burden of atrial fibrillation independently influences the risk of thromboembolism.

Some studies have recently documented that device-detected atrial fibrillation burden, expressed as a percentage of time spent in atrial fibrillation, may have a biological gradient relation with stroke risk and could be combined with the CHA_2_DS_2_-VASc score as a predictor of stroke [4]. However, other studies declare that AF burden has no association with ischemic stroke after adjusting for traditional risk scores (CHA_2_DS_2_-VASc score) [5, 6].

In our cohort, we identified 210 patients with atrial fibrillation by Holter ECG monitoring, and 18 stroke events occurred at a median follow-up of 11.39 months after the Holter ECG monitoring. We did not detect any strong association between AF burden, AF frequency, and subsequent stroke, whereas clinical characteristics-based stratifications such as CHA_2_DS_2_-VASc score were highly predictive of new stroke, with a predictive value of 92% when using CHA_2_DS_2_-VASc score of 4.5 as a cutoff. In this regard, our findings are more consistent with the concept that AF is a marker of an underlying atrial myopathy [7–9] and that once AF occurs, stroke risk can be best predicted by underlying risk factors (as quantified by the CHA_2_DS_2_-VASc score) in patients with atrial fibrillation; conversely, the specific burdens become less relevant.

This difference in findings across studies may lie in the heterogeneity of study cohorts. Most researchers restricted study participation to patients with paroxysmal AF and excluded all other patterns of AF [10], while all patterns of AF patients were included in our cohort. Therefore, AF burden was high in both the stroke group and the control group in our study, which attenuated the difference between the two groups. Meanwhile, compared with those earlier studies, most patients were of advanced age and had multiple comorbidities known to be risk factors for ischemic stroke events in our cohort, with the preponderant high CHA_2_DS_2_-VASc score. These findings further highlight the impact of the clinical characteristics and effectiveness of the CHA_2_DS_2_-VASc scoring system, thus AF burden may become less predictive of stroke among higher risk patients.

In a prospective study of 9850 patients with cardiac implantable electronic device, a strong relationship of temporal proximity of AF with ischemic stroke was found, which was highest in the 5 to 10 days after the episode of AF events (odds ratio 17.4, 95% confidence interval, 5.39–73.1) and no longer elevated by 30 days [11]. It was suggested that rapidly acting anticoagulants should be transient used as a stroke-preventive strategy during onset and offset of paroxysmal or persistent AF. However, a great portion of the subjects had been on medication such as anticoagulant, antiplatelet agents, and statins in our cohort, which reflected good control of ischemic factors and might lead to low and delayed occurrence of endpoint events. Therefore, ischemic stroke occurred at a median of 6.5 months rather than the extremely short-time stroke in the above mentioned studies. AF burden varies in a short time, and its impact on embolism occurs quickly and once the period of atrial fibrillation ends, its impact will disappear rapidly. Our results raise the hypothesis that whether higher CHA_2_DS_2_-VASc increases the risk of relatively long-term stroke while AF burden confers a short-term increase in stroke risk. Further investigation is deserved.

AF was detected by Holter ECG monitoring in our cohort, which was simple, convenient, operable, and more typical of the general AF population. Screening tools such as pacemakers, implantable loop recorders, and even wearable continuous monitors can identify AF better and yield more information related to the variation of AF burden which requires long-term or frequent ECG monitoring [12]. Nonetheless, AF was arbitrarily defined as a HAR event lasting longer than 5–6 min in these studies, and AF may be missed if the onset of AF is short or slow. In addition, on account of AT or frequent premature atrial complexes and far-field *R*-wave over-sensing, there was a 17.3% false-positive rate of cardiovascular implanted electronic devices (CIEDs) detected short period of AF [1, 13]. Such bias could be substantially under control in Holter ECG monitoring because the 12-lead electrocardiogram can detect and analyze atrial waves synchronously, which is helpful to distinguish atrial fibrillation from atrial premature beats, atrial tachycardia, atrial flutter, and pseudo-error. Meanwhile, the cost and invasiveness of these devices limit their widespread adoption, only a few people are implanted with CIEDs, thus it remains unclear whether the conclusions obtained from the small population of CIEDs-detected AF could be directly extrapolated to the practice population, not to mention those without CIEDs. More detailed exploration of the CIEDs-detected AF burden as a novel variable for stroke might need tries in a more comprehensive study cohort with multiple risk factors, and leadless pacemaker may come to the fore, because the overall accuracy of the leadless ICM for AF duration based on the incoherence of the *R*-*R* interval is 98.5% [14].

## 5. Limitation

The core shortcoming of our study is our relatively small sample size and short follow-up of patients with ischemic stroke. We were unable to fully study the low AF burden population because of the small sample size, which may affect the conclusion. Otherwise, this is a single-center cohort study. Therefore, there may be more bias in the results obtained, and a larger study needs to be conducted in a multicenter and large-scale study cohort. Secondly, Holter ECG monitoring can reduce the false positive rate of atrial fibrillation detection, but the prevalence of nonpermanent forms of AF could be largely underestimated compared with more sophisticated screening devices such as pacemakers, implantable loop recorders, and wearable continuous monitors. Thus, reducing the false positive rate of CIEDs detection and promoting its research findings to the public is a key issue to be studied in the future. Wearable devices for similar purposes, such as smartphones, necklaces, watches, and patches with AF sensing technologies, will well beyond the “snapshots” capabilities of traditional monitors.

## 6. Conclusion

In this cohort with Holter ECG monitoring detected AF, AF burden does not significantly impact the subsequent risk of stroke; whereas CHA_2_DS_2_-VASc scoring is still a robust predictor of stroke risk. This illustrates that once AF is detected from Holter ECG monitoring, underlying risk factors appear to be more predictive of subsequent stroke risk than atrial fibrillation burden.

## Figures and Tables

**Table 1:** Baseline characteristics of patients.

Demographics	Stroke group (*n* = 18)	Control group (*n* = 192)	*t*/*χ*^2^	*P* values
Men (%)	12 (66.7)	82 (42.7)	3.82	0.05
Age (y)	75.00 (70.25–83.25)	70.00 (62.50–76.00)	−2.30	0.02
Age (%)				
<65 (y)	2 (11.1)	52 (27.1)	−2.36	0.02
65–75 (y)	6 (33.3)	86 (44.8)		
>75 (y)	10 (55.6)	54 (58.5)		
Hypertension (%)	14 (77.8)	74 (38.5)	10.41	<0.01
Diabetes mellitus (%)	6 (33.3)	24 (12.5)	4.26	0.04
CAD (%)	10 (55.6)	92 (47.9)	0.38	0.54
PAD (%)	8 (44.4)	42 (21.9)	3.46	0.06
Heart failure (%)	12 (66.7)	114 (59.4)	0.37	0.55
Antiplatelet (%)	0 (0.0)	50 (26.0)	4.80	0.03
Statins (%)	10 (55.6)	102 (53.1)	0.04	0.84
Anticoagulant (%)	18 (100%)	118 (61.5)	10.71	<0.01
Anticoagulant efficacy (%)	13 (72.22%)	100 (52.08%)	2.69	0.10
Amiodarone (%)	0 (0.0)	4 (2.1)	0.00	1.00
*ß*-blocker (%)	10 (55.6)	66 (34.7)	3.07	0.08
CHA2DS2-VASc score	6.00 (5.00–7.50)	3.00 (2.00–4.00)	8.90	0.01
CHA2DS2-VASc score (%)			−2.26	0.02
0	0 (0.00)	18 (9.4)		
1	0 (0.00)	26 (13.5)		
≥2	18 (100.00)	148 (77.1)		

*Note*. CHA_2_DS_2_-VASc score included heart failure (1 point), hypertension (1 point) and age 65–74 years (1 point), age ≥75 years (2 points), diabetes (1 point), history of stroke or TIA (2 points), vascular disease (1 point), female (1 point), and the total score is 9. CAD: coronary artery disease; PAD: peripheral artery disease.

**Table 2:** Holter ECG monitoring of patients.

Indexes	Stroke group (*n* = 18)	Control group (*n* = 192)	*t*/*χ*^2^	*P* values
Paroxysmal AF (%)	10 (55.6)	74 (38.5)	1.99	0.16
AF burden	100.00 (88.08–100.00)	100.00 (97.33–100.00)	−0.16	0.87
AF burden (%)			0.46	0.89
**<50%**	2 (11.11)	32 (16.67)		
**50–99%**	2 (11.11)	18 (9.36)		
**>99%**	14 (77.78)	142 (73.96)		
AF frequency	1.00 (1.00–3.50)	1.00 (1.00–2.00)	−1.49	0.14

**Table 3:** Binary logistic regression analysis of stroke event.

Factors	*B*	S.E.	Wald	*P* value	OR	95% CI
Age	−0.26	0.08	10.01	0.01	0.77	0.66–0.91
Hypertension	1.69	1.21	1.95	0.16	5.42	0.51–57.98
Diabetes mellitus	2.05	1.15	3.16	0.08	7.73	0.81–73.63
Antiplatelet	16.58	4149.24	<0.01	1.00	157840044	0.00-
Anticoagulant	−16.945	3378.77	<0.01	1.00	<0.01	0.00-
AF burden	0.02	0.02	0.97	0.33	1.02	0.98–1.08
CHA_2_DS_2_-VASc score	2.72	0.71	14.66	<0.01	15.17	3.77–61.00
Constant	−16.773	4149.24	<0.01	1.00	<0.01	

## Data Availability

Some or all data, models, or code that support the findings of this study are available from the corresponding author upon reasonable request.

## Abbreviations

AF: Atrial fibrillation
AFB: Atrial fibrillation burden
HECG: Holter ECG monitoring
CIEDs: Cardiovascular implanted electronic devices
CAD: Coronary artery disease
PAD: Peripheral artery disease
INR: International normalized ratio.
